# Pre-screening for osteoporosis with calcaneus quantitative ultrasound and dual-energy X-ray absorptiometry bone density

**DOI:** 10.1038/s41598-021-95261-7

**Published:** 2021-08-03

**Authors:** Chia-Chi Yen, Wei-Chun Lin, Tzu-Hao Wang, Guan-Fan Chen, Da-Ying Chou, Dian-Min Lin, Shu-Yuan Lin, Min-Ho Chan, Jia-Ming Wu, Chin-Dar Tseng, Yu-Jie Huang, Tsair-Fwu Lee

**Affiliations:** 1 Department of Orthopedic, Kaohsiung Municipal Min-Sheng Hospital, Kaohsiung, Taiwan; 2 Department of Nutrition, Institute of Biomedical Nutrition, Hung-Kuang University, Taichung, Taiwan; 3 Department of Business Management, National Sun Yat-Sen University, Kaohsiung, Taiwan; 4 Department of Biomedicine Engineering, Chengde Medical University, Chengde, 067000 Hebei China; 5 Medical Physics and Informatics Laboratory of Electronics Engineering, National Kaohsiung University of Science and Technology, Kaohsiung, Taiwan, ROC; 6 Department of Radiation Oncology, Kaohsiung Chang Gung Memorial Hospital and Chang Gung University College of Medicine, Kaohsiung, 83342 Taiwan, ROC; 7 Ph.D. Program in Biomedical Engineering, Kaohsiung Medical University, Kaohsiung, 80708 Taiwan, ROC

**Keywords:** Biocatalysis, Biological techniques, Biophysics, Medical research, Engineering

## Abstract

Calcaneal quantitative ultrasonography (QUS) is a useful prescreening tool for osteoporosis, while the dual-energy X-ray absorptiometry (DXA) is the mainstream in clinical practice. We evaluated the correlation between QUS and DXA in a Taiwanese population. A total of 772 patients were enrolled and demographic data were recorded with the QUS and DXA T-score over the hip and spine. The correlation coefficient of QUS with the DXA-hip was 0.171. For DXA-spine, it was 0.135 overall, 0.237 in females, and 0.255 in males. The logistic regression model using DXA-spine as a dependent variable was established, and the classification table showed 66.2% accuracy. A receiver operating characteristic (ROC) analyses with Youden’s Index revealed the optimal cut-off point of QUS for predicting osteoporosis to be 2.72. This study showed a meaningful correlation between QUS and DXA in a Taiwanese population. Thus, it is important to pre-screen for osteoporosis with calcaneus QUS.

## Introduction

Fractures related to osteoporosis are widely recognized as an important health problem because of their significant morbidity among patients, elevated risk for mortality, and increasing medical, financial, and social costs. Globally, nearly 9 million estimated osteoporosis-related fractures occur annually^1^. In Taiwan, the prevalence of osteoporosis is estimated to be 1.6 million and is quickly increasing. In addition, it is twice as common in women over the age of 50 than in men^2^. According to the World Health Organization (WHO), osteoporosis is defined as “a systemic disease characterized by low bone mass and deterioration of micro-architecture of bone tissue, leading to bone fragility and eventually elevated fracture risk”^3^. The current gold standard for measuring bone mineral density (BMD) is dual X-ray absorptiometry (DXA)^4,5^. The diagnostic criteria of osteoporosis is based on the BMD compared to a reference of Caucasian women aged 20–29, commonly called T-scores, which must be lower than 2.5 standard deviations (SDs)^6^. However, this method is costly, instrument-based, involves ionizing radiation, and requires highly trained operators to minimize error, possibly leading to the low use of DXA assessment as a screening tool^7^. These disadvantages may explain the underdiagnosis of osteoporosis in Taiwan and globally^8^.

Calcaneal quantitative ultrasound (QUS) is an alternative approach for assessing bone health and identifying osteoporosis. Since its introduction in 1984, QUS has gained popularity in recent years for being cheaper, portable, free of ionizing radiation, and easier to handle^9^. QUS assesses bone health by measuring the propagation of ultrasound waves, a frequency that exceeds the normal auditory range of humans (> 20 kHz), at varying frequencies. Two parameters are commonly generated by QUS, namely, the speed of sound (SOS) and the velocity of sound (VOS) and broadband ultrasound attenuation (BUA)^10^. The SOS refers to the transmission time of the wave through the length of body parts. Broadband attenuation occurs when sound waves pass through soft tissue and bone and energy is absorbed. There is a combined score called the Stiffness Index (SI), which combines the velocity and attenuation using different algorithms. The combination of these variables to QUS value was calculated by proprietary software. Calcaneus is the most studied and only recognized skeletal site for QUS assessment because of the high percentage of trabecular bone and two lateral surfaces that facilitate ultrasound waves and provide easy accessibility^11^.

The use of calcaneal QUS as a diagnostic method for osteoporosis compared to the current gold standard (DXA) has been evaluated in several studies. Many approaches have been evaluated including bone health, prediction of fracture risk, and correlation with T-scores^12^. For every SD decrease in the QUS-measured variable, fracture risk for the hip and spine increase by two-fold, which is comparable to DXA^13,14^. However, there is little consensus for using QUS as a diagnostic tool for osteoporosis compared to DXA. The interpretation of QUS result in assessment of bone quality and related medical treatment remains to be elucidated. A meta-analysis concluded that there is no definite threshold for QUS when identifying osteoporosis compared to DXA T-scores^15,16^.

As a prescreening tool for osteoporosis, the goal is to classify low-risk and high-risk patients and to ensure DXA examination for the high-risk patients. This should increase the accessibility of DXA and improve the diagnosis rate for osteoporosis. For that purpose, a high-sensitivity examination is required as well as a triage approach that can identify two cutoffs at device-specific sensitivity and specificity levels of 90% or 95%^12^. This approach could determine the correlation between the QUS and DXA value, allowing the assessment of the benefit of calcaneal QUS and making it possible to establish a cutoff where follow-up DXA (spine and hip) is required.

It is also important to establish cutoff levels that can rule in or rule out osteoporosis. Although several studies have compared values between calcaneal QUS and DXA, few studies have been conducted on Asian populations. We performed a large population-based study in Taiwan to explore the relationship between cutoff and accuracy of calcaneal QUS to identify elderly (age > 60 y/o) with low QUS levels (T ≤ − 2.0) and to investigate the ability of QUS to reduce the number of patients who require referral for DXA at cutoffs corresponding to 90–95% certainty levels. In this study, a multivariate logistic regression model using QUS as a covariate was explored for the feasibility of using QUS as a diagnostic tool in osteoporosis.

## Materials and methods

The study protocols were approved by Kaohsiung Veterans General Hospital institutional review board (KSVGH20-CT7-13). This study was conducted from January 2020 to March 2020 during the annual municipal elderly health examination in Kaohsiung Municipal Min-Sheng Hospital. Calcaneal QUS was performed on every elderly subject who participated in the health examination. For subjects that met two criteria (age ≥ 65 years and calcaneal QUS ≤ − 2.0), DXA examination was arranged for further evaluation of osteoporosis. In some subjects with QUS > − 2.0, DXA was performed if osteoporosis was suspected, based on clinical grounds. Both spine and hip DXA were recorded as T-score of BMD. Demographic data including age, sex, height, body weight, medical history, fracture history, and potential secondary causes of osteoporosis were recorded. A total of 772 patients were enrolled and subjects who were diagnosed with osteoporosis and were under treatment or those with old fractures of the calcaneus were excluded. The study was approved by the local and regional ethics committees and was conducted in accordance with the Code of Ethics (Declaration of Helsinki). Informed consent was waived. According to WHO criteria for the classification of osteoporosis, a T-score of − 1.0 and greater was considered normal bone density, a T-score between − 1.0 and − 2.5 was considered low bone density (osteopenia), and a T-score of − 2.5 and less was defined as osteoporosis.

The QUS was measured using a Pegasus device (BeamMed Ltd., Tel Aviv, Israel), which is designed to measure the SOS (m/s) of ultrasonic waves that travel longitudinally along the bones at a center frequency of 1.25 MHz. The machine uses gel as a coupling agent between the probe and skin. QUS can be measured at either the left or right calcaneus. The device was calibrated before each data collection using a verification phantom provided by the manufacturer. The QUS T-score was calculated according to the normative data derived from a sex- and age-matched Asian population, provided by the manufacturer. The QUS scans were performed by two independent physicians. Patients with calcaneal QUS values under − 2.0 SDs are referred for a DXA scan. BMD, which is expressed in grams per centimeter squared (g/cm^2^), was measured using the DXA technique. The DXA machine was calibrated daily using a spine phantom supplied by the manufacturer prior to measurements. Then the subjects were positioned and instructed to stay motionless throughout the scan. Each complete scan took approximately 15 min. BMD T-scores were calculated based on an Asian age- and sex-matched population provided by the DXA manufacturer. Measurements were made to ensure coverage of the lumbar and hip regions. The average, as well as individual, vertebral, and hip BMD were recorded.

### Statistical analyses

Data are presented as means ± SDs for numerical variables and frequency or percentage for categorical variables. Correlation analyses were performed using a two-tailed Pearson correlation coefficient with a significance level of p < 0.05. A receiver operating characteristic (ROC) curve and the area under the curve (AUC) were calculated to assess the discrimination power of QUS with regard to the gold standard of DXA. The optimal cut-off value for calcaneal QUS for classification of bone status was based on Youden’s J statistics as Eq. 1^17^:

1 $${\text{J }} = {\text{ sensitivity }} + {\text{ specificity }}{-}{ 1}{\text{.}}$$

All statistical analyses were conducted using Statistical Package for Social Sciences (SPSS) version 26.0 for Windows (SPSS Inc., Chicago, IL).

### Ethical statement

The requirement for informed consent was waived by Kaohsiung veterans gerneral hospital institutional review board (KSVGH20-CT7-13) given the retrospective nature of the study.

## Results

**Figure 1:**
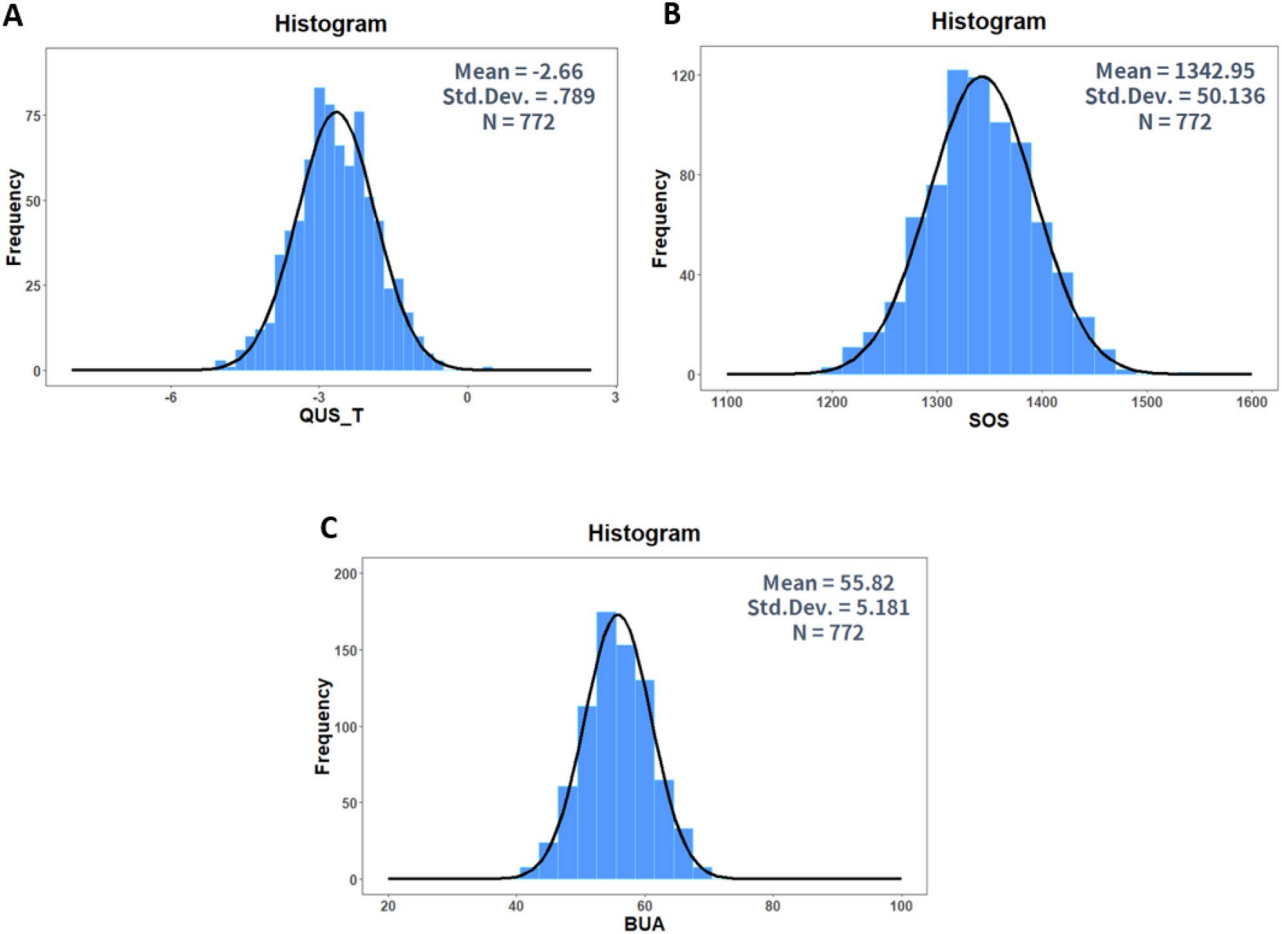
(**A**) Histogram of calcaneus QUS T-score data. (**B**) Histogram of the SOS data by QUS machine. (**C**) Histogram of the BUA data by QUS machine. Figure linework and aesthetics were created in R 3.6.2; link to software homepage. *QUS_T* quantitative ultrasonography T-score, *SOS* speed of sound, *BUA* broadband ultrasound attenuation.

**Figure 2:**
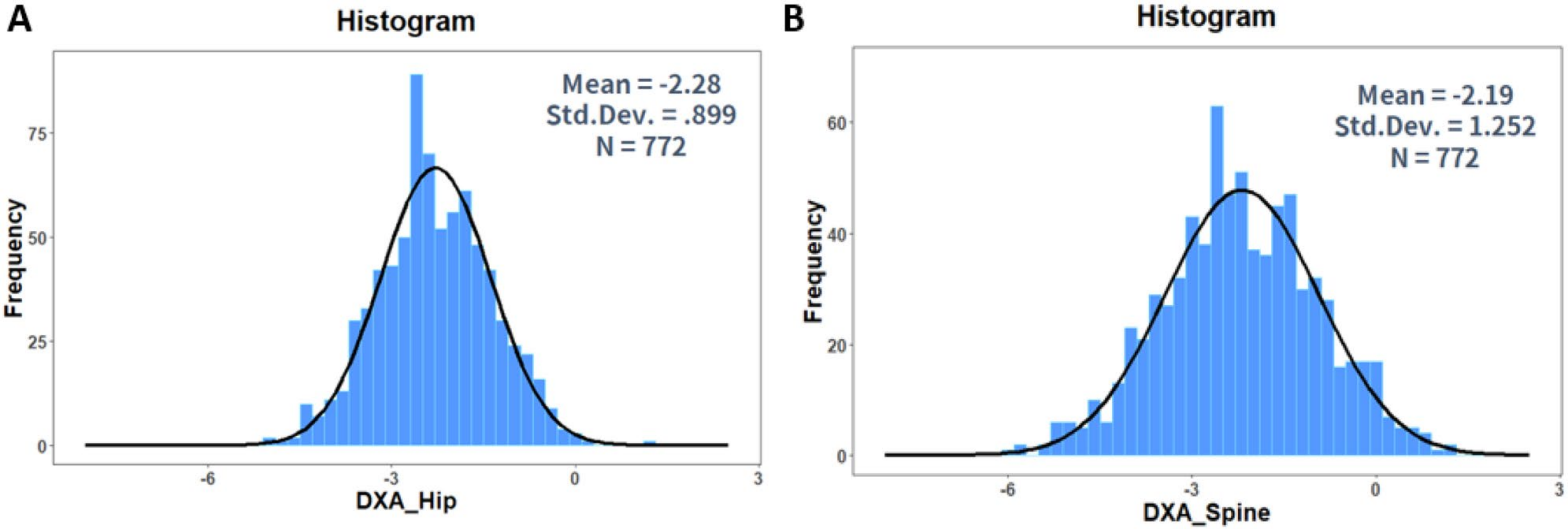
(**A**) Histogram of DXA-Hip data. (**B**) Histogram of DXA-Spine data. Figure linework and aesthetics were created in R 3.6.2; link to software homepage. *DXA-Hip* dual-energy X-ray absorptiometry of Hip, *DXA-Spine* dual-energy X-ray absorptiometry of Spine.

**Figure 3:**
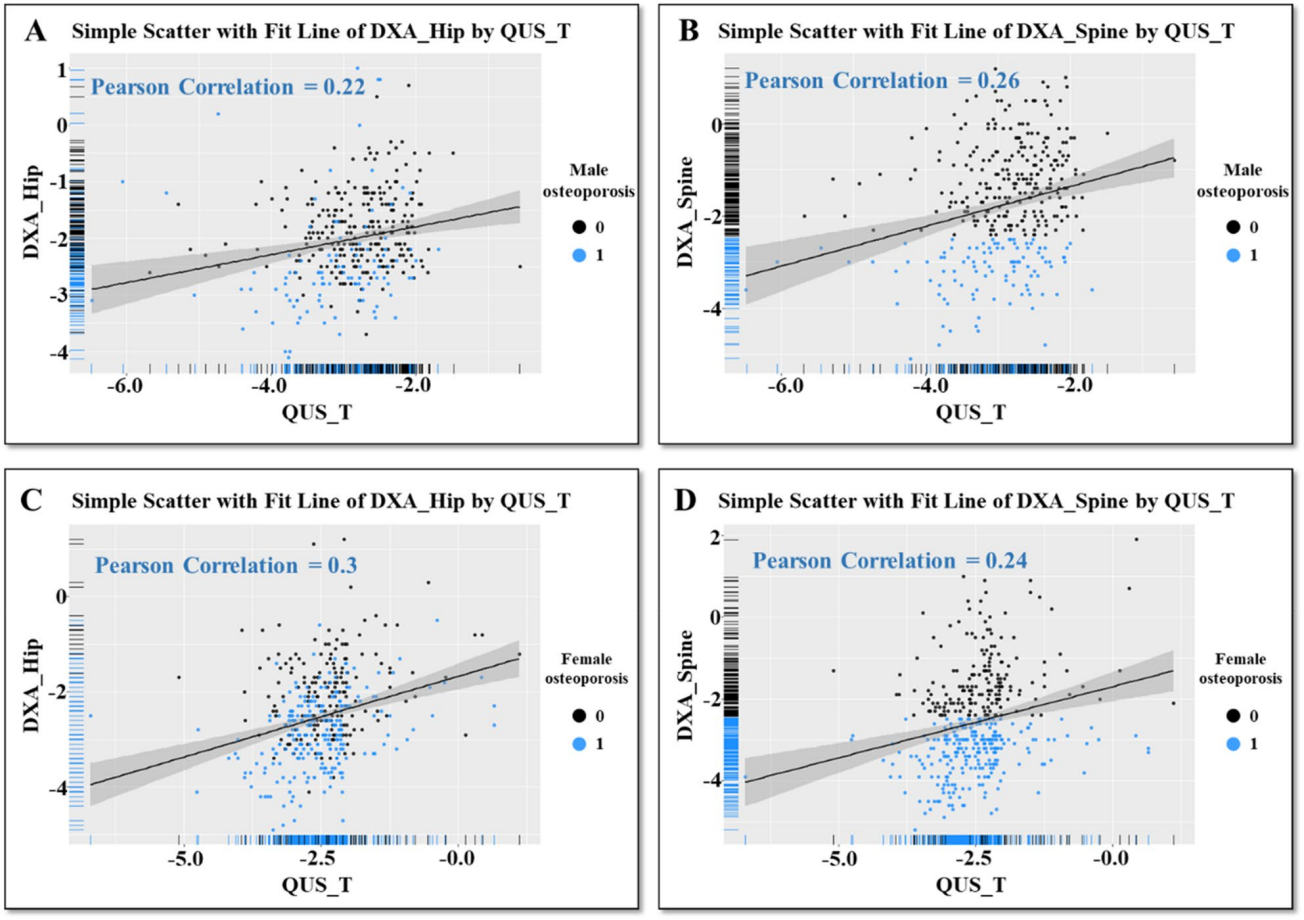
(**A**) Scatter plot and regression line of calcaneus QUS_T and DXA-Hip of male osteoporosis. (**B**) Scatter plot and regression line of calcaneus QUS_T and DXA-spine of male osteoporosis. (**C**) Scatter plot and regression line of calcaneus QUS_T and DXA-Hip of female osteoporosis. (**D**) Scatter plot and regression line of calcaneus QUS_T and DXA-spine of female osteoporosis. Figure linework and aesthetics were created in R 3.6.2; link to software homepage. *QUS_T* quantitative ultrasonography T-score, *DXA-Hip* dual-energy X-ray absorptiometry of Hip, *DXA-Spine* dual-energy X-ray absorptiometry of Spine.

This study consisted of 772 patients, including 352 men (45.6%) and 420 women (54.4%). Mean age was 72.9 ± 6.3 (range 48–99) years. In females, osteoporotic subjects were aged 72.2 ± 6.0 years, while non-osteoporotic subjects were aged 71.4 ± 6.3 years. Regarding males, osteoporotic subjects were aged 74.7 ± 7.0 years, while non-osteoporotic subjects were aged 73.8 ± 5.8 years. QUS and DXA values are shown in Table 1. Histogram data for QUS, SOS, and BUA are shown in Fig. 1, histogram data for DXA-hip and DXA-spine are given in Fig. 2, and scatter plots with regression lines are shown in Fig. 3. The correlation coefficient between QUS and DXA-hip was 0.171 (p < 0.001); that between QUS and DXA-spine was 0.135 (p < 0.001). These values were 0.298 and 0.237, respectively, in females and 0.216 and 0.255 in males. All results were significant.

**Table 1:** Descriptive statistics of QUS and DXA variable.

	N	Minimum	Maximum	Mean	Std. deviation
QUS_T	772	− 6.70	1.11	− 2.6596	0.78859
DXA_Hip	772	− 4.90	1.20	− 2.2846	0.89920
DXA_Spine	772	− 5.20	1.90	− 2.1874	1.25186

*QUS_T* quantitative ultrasonography T-score, *DXA-Hip* dual-energy X-ray absorptiometry of Hip, *DXA-Spine* dual-energy X-ray absorptiometry of Spine.

**Figure 4:**
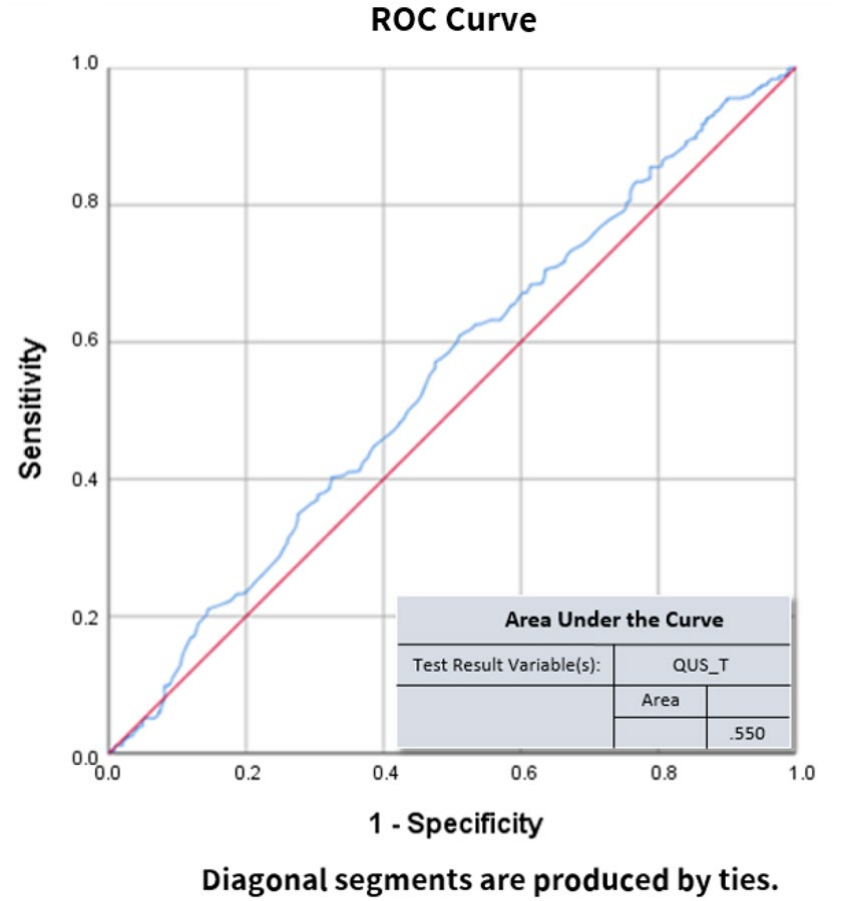
ROC curve analysis using QUS as predictive variable. The test result variable(s) = QUS_T has at least one tie between the positive actual state group and the negative actual state group. Statistics may be biased. Figure linework and aesthetics were created in R 3.6.2; link to software homepage. *ROC* *curve* receiver operating characteristic curve, *sensitivity* measures the proportion of actual positives that are correctly identified, *1-specificity* missed diagnosis rate, *QUS_T* quantitative ultrasonography T-score.

**Figure 5:**
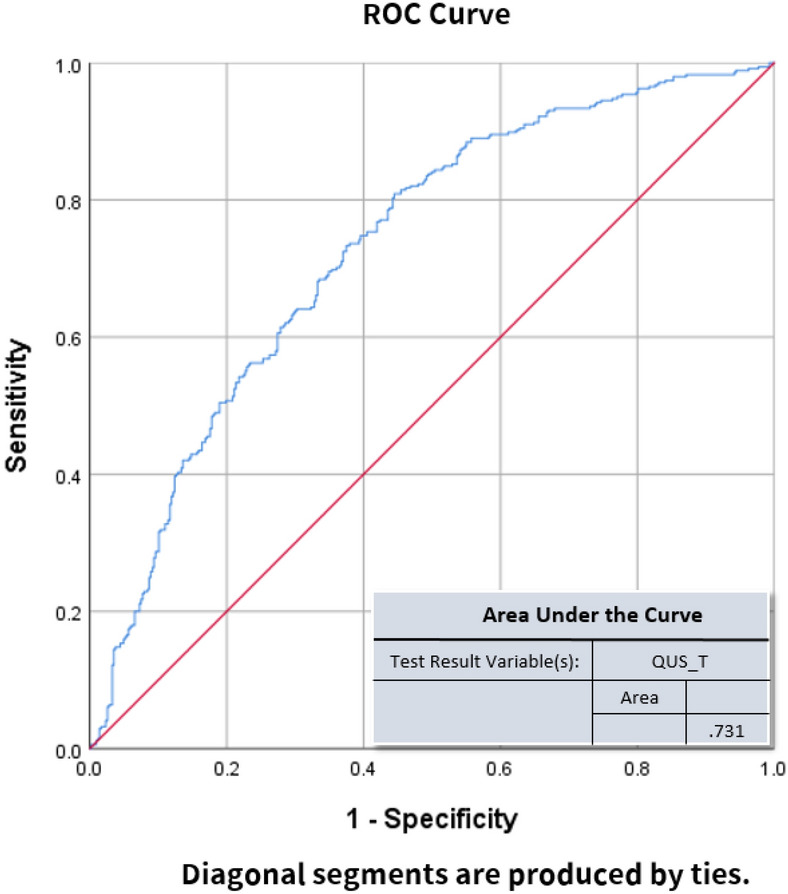
The ROC curve analysis of probability calculated from logistic regression model as predictive variable. Figure linework and aesthetics were created in R 3.6.2; link to software homepage. *ROC* *Curve* receiver operating characteristic curve, *QUS_T* quantitative ultrasonography T-score, *sensitivity* measures the proportion of actual positives that are correctly identified, *1-specificity* missed diagnosis rate.

To evaluate the discriminating power based on a single QUS variable to predict osteoporosis, ROC analyses were performed with the ground truth set as DXA-spine T-score < − 2.5. Results of the ROC analyses are shown in Fig. 4. To increase the discriminating power, multivariate logistic regression was performed with more independent variables. Age, sex, body weight, height, body mass index (BMI), SOS, BUA, and QUS-T were defined as explanatory variables to predict the osteoporosis status defined by DXA-spine. Logistic regression coefficients are shown in Table 2. Log-odds coefficients with Wald statistics show that the variables of sex, body weight (BW), body height (BH), and BMI have statistical significance (p < 0.05). The correlation between variables are also displayed in Table 2. The logistic regression model had 66.2% accuracy, 67.2% sensitivity, and 64.9% specificity. Using the predicted probability obtained from the logistic regression model, the ROC curve was recalculated and is shown in Fig. 5. This more sophisticated logistic regression model had an AUC of 0.731.

**Table 2:** Descriptive statistics of QUS and DXA variable.

Step	− 2 Log likelihood	Cox and Snell R^2^	Nagelkerke R^2^							
**Model Summary**										
1	941.230^a^	0.144	0.193							

Observed			Predicted True_state		Percentage correct					
			0	1						
**Classification table**^**b**^										
Step 1	True_state	0	287	140	67.2					
		1	121	224	64.9					
	Overall percentage				66.2					

		Log-odd coefficient	Standard error	Wald statistics	df	p value	Odds probability	95% Confidence interval for odds		
								Lower	Upper	
**Variables in the equation**										
Step 1^c^	Age	0.010	0.013	0.542	1	0.461	1.010	0.984	1.037	
	Sex (1)	1.485	0.451	10.825	1	0.001	4.413	1.823	10.687	
	BW	− 0.322	0.084	14.835	1	0.000	0.725	0.615	0.854	
	BH	0.210	0.063	11.126	1	0.001	1.234	1.091	1.396	
	BMI	0.714	0.196	13.322	1	0.000	2.043	1.392	2.998	
	BUA	0.091	0.098	0.866	1	0.352	1.096	0.904	1.328	
	SOS	0.001	0.002	0.454	1	0.500	1.001	0.998	1.004	
	QUS_T	− 1.035	0.655	2.497	1	0.114	0.355	0.099	1.282	
	Constant	− 42.139	12.431	11.491	1	0.001	0.000			

		Constant	Correlation matrix							
			Age	Sex (1)	BW	BH	BMI	BUA	SOS	QUS_T
Step 1	Constant	1.000	− 0.107	− 0.570	0.770	− 0.789	− 0.772	− 0.571	− 0.203	0.570
	Age	− 0.107	1.000	0.099	− 0.004	0.048	0.011	− 0.046	0.083	0.052
	Sex (1)	− 0.570	0.099	1.000	0.019	0.040	− 0.015	0.868	0.012	− 0.881
	BW	0.770	− 0.004	0.019	1.000	− 0.979	− 0.997	0.008	− 0.090	− 0.008
	BH	− 0.789	0.048	0.040	− 0.979	1.000	0.978	− 0.012	0.084	0.010
	BMI	− 0.772	0.011	− 0.015	− 0.997	0.978	1.000	− 0.008	0.091	0.008
	BUA	− 0.571	− 0.046	0.868	0.008	− 0.012	− 0.008	1.000	− 0.072	− 0.986
	SOS	− 0.203	0.083	0.012	− 0.090	0.084	0.091	− 0.072	1.000	0.062
	QUS_T	0.570	0.052	− 0.881	− 0.008	0.010	0.008	− 0.986	0.062	1.000

*BW* body weight, *BH* body height, *BMI* body mass index, *BUA* broadband ultrasound attenuation, *SOS* speed of sound, *QUS_T* quantitative ultrasonography T-score, *df* degree of freedom.

^a^Estimation terminated at iteration number 5 because parameter estimates changed by less than 0.001.

^b^The cut value is 0.500.

^c^Variable(s) entered on step 1: Age, Sex, BW, BH, BMI, BUA, SOS, QUS_T.

**Figure 6:**
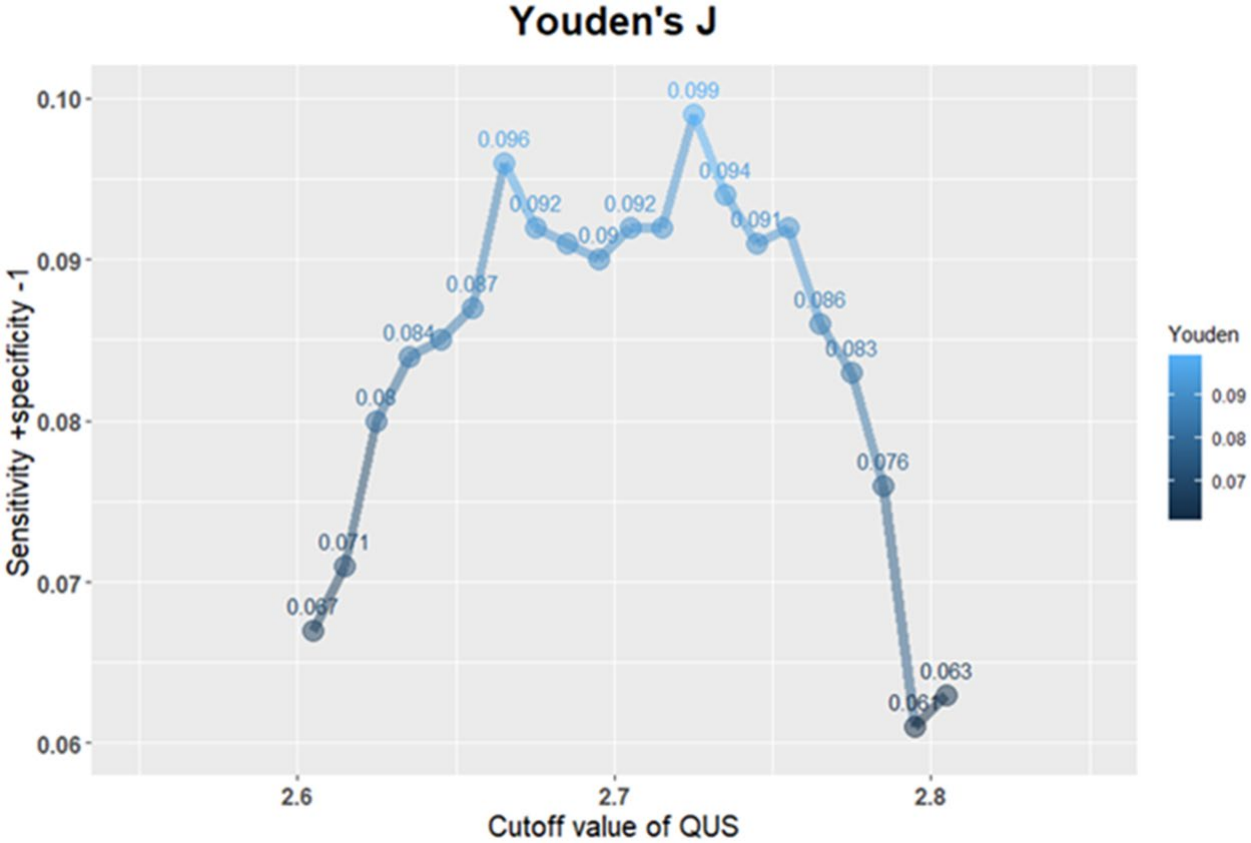
ROC curve analysis with coordinates for calculating the Youden’s index. Figure linework and aesthetics were created in R 3.6.2; link to software homepage. *Youden's* *J* single statistic that captures the performance of a dichotomous diagnostic test, *QUS* quantitative ultrasonography.

To identify the optimal cutoff value of QUS for the diagnosis of osteoporosis, the Youden’s Index was adopted, using absolute values. The sensitivity and specificity are shown in Table 3. Youden’s Index is the sum of sensitivity and specificity minus one and 2.725 was established as the optimal cutoff value, as shown in Fig. 6.

**Table 3:** Corresponding sensitivity and specificity level with different cutoff values of QUS.

Cut-off values	Sensitivity	Specificity	Youden's J
− 2.6050	0.545	0.522	0.067
− 2.6150	0.542	0.529	0.071
− 2.6250	0.536	0.543	0.080
− 2.6350	0.533	0.550	0.084
− 2.6450	0.530	0.555	0.085
− 2.6550	0.525	0.562	0.087
− 2.6650	0.525	0.571	0.096
− 2.6750	0.516	0.576	0.092
− 2.6850	0.510	0.581	0.091
− 2.6950	0.504	0.585	0.090
− 2.7050	0.501	0.590	0.092
− 2.7150	0.493	0.600	0.092
− 2.7250	0.490	0.609	0.099
− 2.7350	0.478	0.616	0.094
− 2.7450	0.472	0.618	0.091
− 2.7550	0.467	0.625	0.092
− 2.7650	0.461	0.625	0.086
− 2.7750	0.455	0.628	0.083
− 2.7850	0.443	0.632	0.076
− 2.7950	0.429	0.632	0.061
− 2.8050	0.426	0.637	0.063

Cutoff value = optimal decision threshold, sensitivity = measures the proportion of actual positives that are correctly identified, 1-specificity = missed diagnosis rate, Youden's J = single statistic that captures the performance of a dichotomous diagnostic test.

## Discussion

We assessed the validity of QUS as a screening method for osteoporosis in a Taiwanese population. We found a significant correlation between QUS and DXA T-scores in both the hip and spine (p < 0.05). The correlation coefficient between QUS and DXA-hip was 0.174, higher than that between QUS and DXA-spine (0.133). This may originate from the fact that the calcaneus and femoral neck belong to the lower limbs, sharing similar bony architectures. However, the DXA-spine remains the first-choice diagnostic method for osteoporosis in clinical practice. Therefore, once osteoporosis is suspected based on calcaneus QUS, further DXA examination is required to confirm the diagnosis of osteoporosis. Although the correlation between QUS and DXA-hip, and DXA-spine was not high (0.174 and 0.133, respectively) because of large sample sizes, the existence of positive correlation implied feasibility of regression models.

The correlations between QUS and DXA also differed within each sex. For example, in females, it was 0.298 for DXA-hip but 0.237 for DXA-spine; in males, the values were 0.216 and 0.255, respectively. This difference arises from several reasons, one of them is skin contact surface with the ultrasound transducer over the calcaneus region. The subcutaneous fat component is more abundant in females, providing a more consistent medium for conducting the ultrasound wave. In contrast, subcutaneous fat levels are lower in males, and more ultrasound energy is dissipated at various tissue junctions, resulting in inconsistent QUS measurements. In this study, about 31 outlier cases were identified, all with abnormally high SOS values. Males made up 23 of these outliers (about 67.5%). This phenomenon also can be explained by skin contact surface heterogeneity of the calcaneus region between both sexes.

The process of DXA acquisition takes approximately 20 min for study completion. Besides the consideration of ionizing radiation, the patient’s position also requires trained operators to minimize errors. On the contrary, QUS acquisition takes only 3 s. Automatic ultrasound probes can lead to consistent results when the subject’s foot is at the right position. QUS is quicker, and requires less trained operators.

The main reason why the QUS cannot be a standard method for osteoporosis assessment as permitted by WHO is due to the many uncertainty factors during the measurement process. However, the standard deviation in Table 1 shows more data consistency of QUS than DXA (0.78859 vs. 1.25186). QUS only measures the calcaneus, which is mainly composed of trabecular bone. Associated cortical sclerosis and tendon calcification are rare for measurement. DXA measures the lumbar spine and proximal femur, and in this region, cortical sclerosis and tendon calcification is often seen. This bony structural difference may explain the standard deviation observed in Table 1.

The physical basis of QUS is measured by two ultrasound parameters on the calcaneus region, i.e., the SOS and BUA. The SOS and BUA are combined to calculate the T-score of QUS, as predefined by the manufacturer. The histogram data for QUS, SOS, and BUA are shown in Fig. 1. The data are similar. However, the histogram data for DXA-hip and DXA-spine show a right-skewed (log-normal) distribution (Fig. 2)^18^. This may have caused the discrepancies between QUS and DXA.

Figure 3 shows the results of regression analyses, including scatter plots of QUS and DXA-Hip and DXA-spine data. The plots are concentrated around the − 2.5 region, with fewer cases at QUS > − 1.0. The reason for this is due to the inclusion criteria during the annual health examination; only subjects with QUS − 2.0 were chosen for further DXA examination. This results in a highly concentrated data distribution; therefore, we adopted some cases with higher QUS values to study the regression line.

Figure 4 shows the sensitivity and specificity of using only QUS as a predictive variable for osteoporosis, according to ROC analyses. The AUC was 0.55 not far from the reference line which is AUC of 0.5. To increase discriminative power, we ran a multivariate logistic regression. The predictive variables included age, sex, body weight, height, BMI, BUA, and SOS. The logistic regression model had a sensitivity of 67.2% and a specificity of 64.9%, with an overall accuracy of 66.2%. Female had a 4.4-fold higher odds ratio than males with osteoporosis. Another significant predictive variable was QUS (where a lower value = osteoporosis more likely). Due to the improved performance of this more sophisticated logistic regression, the probability predicted by this model can be used as a new test variable for ROC analyses. These results suggest that using a more sophisticated model will increase the discrimination power for osteoporosis. The weighting of individual factors can also be examined based on the results of the logistic model described in Table 2.

**Figure 7:**
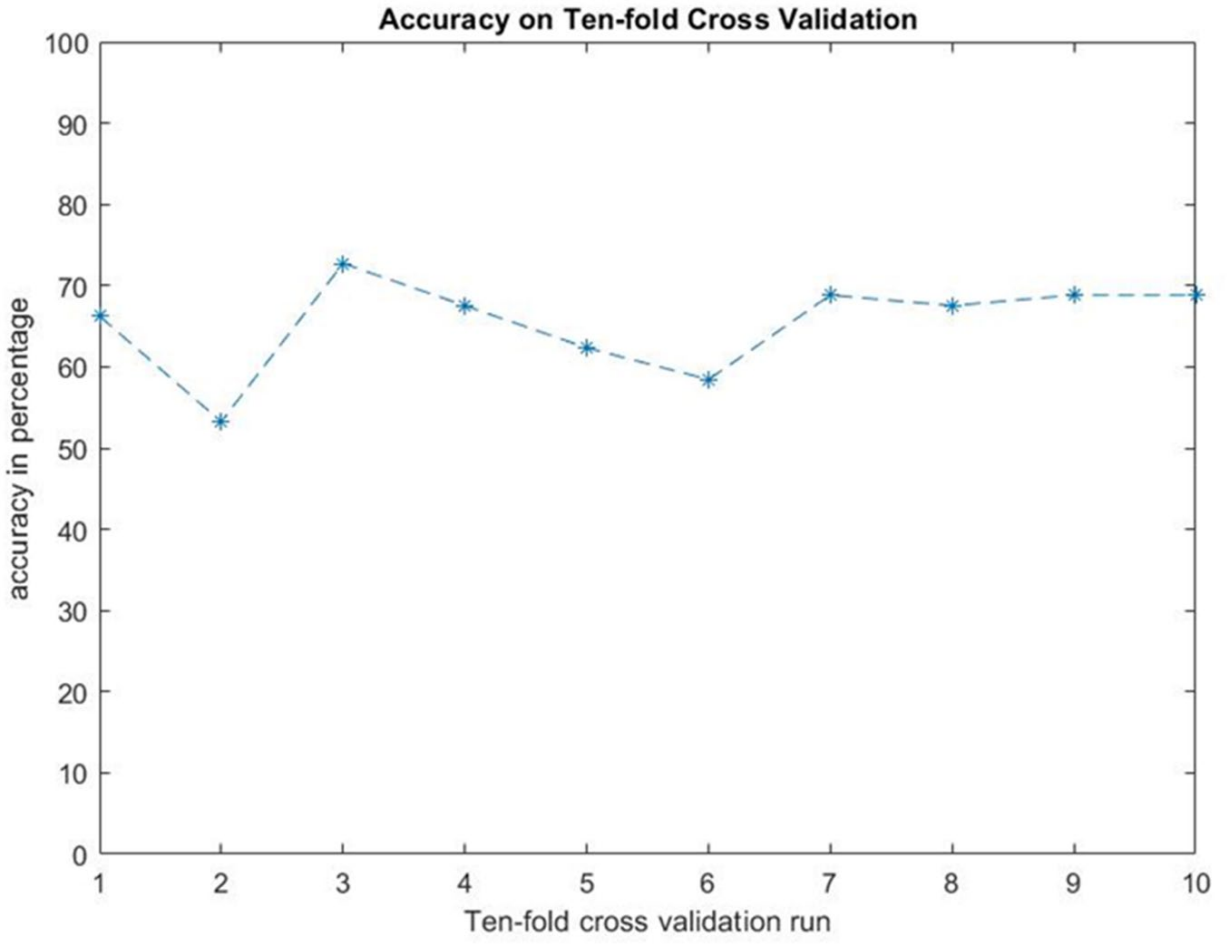
Ten-fold cross validation of the logistic regression model.

The accuracy of this multivariate logistic regression model was 66.2%. This result was obtained from maximum likelihood estimation (MLE) on the logistic model. The error rate comes from regression residue which cannot be eliminated due to finite parameter number in the equation. In order to demonstrate the generalizability of this model, ten-fold cross validation was performed using the MATLAB programming language. The correctness of MATLAB output is checked with SPSS. For each run of cross validation, 90% data is used as the training set, the regression coefficient is obtained, and the remaining 10% data is used as the testing set. Finally, the accuracy rate of model in each turn is recorded, as shown in Fig. 7. The mean value of accuracy is 65.5%.

The WHO defined the status of osteoporosis as a DXA T-score < − 2.5. In this study, the ground truth of the examined osteoporotic subjects was established using the DXA T-score of either the spine or hip. The lumbar vertebrae T-score was recorded as the average value for the L1–L5 lumbar segments. This process automatically omits segments that are abnormal, such as those with a compression fracture. If all the lumbar vertebrae are unavailable for DXA judgement, such as in cases with spinal instrumentation and fusion, the hip DXA T-score will be adopted. DXA-hip value focus only the femoral neck area as the sampling region. This is compatible with criteria used in clinical situations.

To determine the optimal cutoff value for QUS data, Youden’s J statistic was adopted. The Youden Index seeks the maximum value in an ROC curve (specificity + sensitivity − 1). As shown in Fig. 6, the maximum value corresponded to a QUS of 2.725. Therefore, when the QUS is used alone as a predictive variable of osteoporosis, − 2.725 is automatically the optimal threshold for defining disease status. This value is similar to the criteria established by the WHO when judging DXA results.

On the other hand, the Younden index as aforementioned is only one way of selecting the optimal cutoff value in the ROC curve. In the Younden approach, sensitivity and specificity are of equal weighting. There exist other criteria for selection of the optimal cutoff value; for instance, using the Euclidean distance method to find the closest point to the top left corner point seems to result in a sensitivity of ~ 0.57 and specificity of ~ 0.52, and a threshold closer to zero. Based on these criteria, it will result in a more sensitive test and reduce the chance of under-diagnosis. Sometimes it is more important to find the osteoporotic subjects than to rule out negative subjects.

Although DXA is the gold standard for diagnosis of osteoporosis, quantitative computed tomography (QCT) is also utilized for measuring BMD in some circumstances. In the future, it is of interest to conduct a head-to-head randomized clinical trial to further assess the relative efficacy of QUS and DXA.

There were two main limitations of this study. First, during the annual health examination for elderly patients, we did not collect data on subjects younger than 65 years old. Second, for cases that did not meet the inclusion criteria (age ≥ 65 years old and calcaneal QUS ≤ − 2.0), the DXA scan was performed only for a small portion of patients. Therefore, we lack data on patients with higher QUS values (Supplementary Information).

## Conclusions

In conclusion, QUS is a feasible and noninvasive method for measuring bone status in elderly populations in Taiwan. Due to the significant correlation between QUS and DXA, the potential for QUS as a pre-screening tool has been well explored. Although QUS is not the gold standard for diagnosis of osteoporosis, because of convenience and low cost, it is an attractive alternative to conventional DXA in some situations. Multivariate logistic regression models have more discriminative power than single variable model using the QUS. Furthermore, the optimal Younden’s Index cutoff value for QUS to confirm osteoporosis is − 2.725.

## Supplementary Information

Supplementary Information 1.

Supplementary Information 2.

## Data Availability

The data supporting the results of this article are included within this manuscript and supplementary.
