# Supplementary material for: Pre-screening for osteoporosis with calcaneus quantitative ultrasound and dual-energy X-ray absorptiometry bone density
Source: Sci Rep. 2021 Aug 3;11:15709. doi: 10.1038/s41598-021-95261-7 (PMC8333105; doi:10.1038/s41598-021-95261-7)
Supplement: Supplementary file 2 — Supplementary Information 2. [file 41598_2021_95261_MOESM2_ESM.docx]

% MATLAB code

clear;

close all;

[NUM,TXT,RAW]=xlsread('Data.xlsx');

D=double(NUM(:,1:8)); %D is 770x8

Y=categorical(NUM(:,9)); % Y is ground true, 770x1

Pred=double(NUM(:,10)); % Pred is 770x1 vector: original model using SPSS 770 based

B=zeros(10,9);

numberOfZeros=zeros(10,1);

percentage=zeros(10,1);

for i=1:10

Dtrain=D;

starting=1+ 77*(i-1);

ending=77*i;

Dtrain(starting:ending, : )=[]; % Ytrain is 693 x 8

Ytrain=Y;

Ytrain(starting:ending, : )=[]; % Ytrain is 693 x 1

Dtest=D(starting:ending, :); %Dtest is 77x8

Ytest=Y(starting:ending, :); %Ytest is 77x1

PrTest=Pred(starting:ending,:); %PrTest is 77x1,

Truth=NUM(starting:ending,9); %Truth is osteoporosis status,

b= mnrfit(Dtrain,Ytrain); % b is 9x1, coefficient the first one

B(i,:)=b';

D1=ones(77,9);

D1(:,2:9)=Dtest(:,:); %D1 is extended data, 77x9

z=D1*b; % Z is 77x1 vector

p=1./(1 + exp(z)); %p is 77x1 vector

r=round(p); %r is p rounded to integer, 77x1

% rPre=round(PrTest); % 77x1, model from using SPSS

diff=r-Truth;

numberOfZeros(i,1)= sum(diff(:)==0);

percentage=numberOfZeros./0.77;

end

plot(percentage,'--*');

title('Accuracy on Ten-fold Cross Validation');

xlabel('Ten-fold cross validation run');

ylabel('accuracy in percentage');

axis([1 10 0 100]);

meanValue=mean(percentage);
